# Use of amantadine in traumatic brain injury: an updated meta-analysis of randomized controlled trials

**DOI:** 10.3389/fneur.2024.1444623

**Published:** 2025-01-21

**Authors:** João Félix, Luísa Araújo, Antônio Henriques, Ana Pereira, Saul Carneiro

**Affiliations:** School of Medicine, Institute of Medical Sciences, Federal University of Pará, Belém, Brazil

**Keywords:** amantadine, traumatic brain injury, neurotrauma, meta-analysis, placebo

## Abstract

**Introduction:**

Amantadine has been shown to accelerate cognitive and functional brain recovery after cerebrovascular accidents. However, the efficacy of this drug in TBI patients remains poorly defined.

**Methods:**

We performed a systematic review and meta-analysis of randomized trials (RCTs) evaluating the effects of amantadine in TBI patients. The Cochrane, Embase, and PubMed databases were systematically searched for trials published up to March 24, 2024. Data from previous RCTs were extracted and quality assessed according to Cochrane recommendations. Means and standard deviations with 95% confidence intervals were aggregated across studies. The primary outcomes assessed were Glasgow Coma Scale (GCS), Mini Mental State Examination (MMSE) and the Disability Rating Scale (DRS).

**Results:**

From 1,292 database results, 6 studies with 426 patients were included, of which 205 received amantadine (48.12%). The Glasgow Coma Scale score on day 7 (MD 1.50; 95% CI 0.08–2.92; *p* = 0.038; *I*^2^ = 68%) was significantly higher in patients treated with amantadine than those treated with placebo. The Mini Mental State Examination (MD 3.23; 95% CI 0.53–5.94; *p* = 0.019; *I*^2^ = 0%) was also better in patients treated with amantadine. No significant differences in Disability Rating Scale, day 3 GCS, Glasgow Outcome Scale (GOS), length of hospital stay, or duration of mechanical ventilation were observed between amantadine and placebo groups.

**Conclusion:**

In our analysis, TBI patients benefit from the use of amantadine in the day 7 GCS score and show better results in the MMSE test, but placebo patients benefit from not using amantadine in the DRS between weeks 3 and 4. No other statistically significant results were found related to the use of this medication.

**Systematic review registration**: https://www.crd.york.ac.uk/prospero/display_ record.php?ID=CRD42024538110, CRD42024538110.

## 1 Introduction

Traumatic Brain Injury (TBI) is an important cause of death and disability worldwide (1). The global annual incidence of TBI was estimated at 27 million (2). The condition progresses to loss of consciousness and often leads to permanent neurocognitive sequelae for the patient due to the different forms of possible neurological injury, such as extrinsic compression from mass lesion, contusion, diffuse axonal injury [DAI] (3).

There are several general measures for the management of patients with TBI, including euvolemia, 30° head position, use of prophylactic anticonvulsants, and correction of hypoxemia, hypoventilation, and hypotension (4). In addition to these measures, several medications have been used to directly improve the level of consciousness in TBI patients, such as corticosteroids, progesterone, and erythropoietin (5–8). One of these drugs, amantadine, is a neurostimulant commonly used to treat Parkinson’s disease. Drugs with dopaminergic effects provide wakefulness and improve nervous system damage by stimulating the reticular activating system (9, 10). The drug is also an antagonist of the N-methyl-D-aspartate receptor (NMDARs) and has anti-inflammatory effects on the brain (11). The drug has been reported to contribute to neurocognitive improvements in patients with TBI, both its direct agonistic effect on dopamine receptors and its NMDARs antagonist effect (10–12).

Two studies (13, 14) found favorable results for amantadine in the first 4 weeks of treatment when the drug was used for less than 1 month, whereas one randomized controlled trial (10) found no benefit with the use of amantadine compared to placebo regardless of the time of administration. In contrast, Giacino and Meythaler (12, 13) found an improvement in Disability Rating Scale (DRS) when patients are treated with amantadine versus placebo. Therefore, there is still disagreement in the literature regarding the use of amantadine in TBI. Although none of the previous meta-analyses analyzed patients’ cognitive improvement by each functional outcome scale. Similarly, there are still no analyses comparing mean length of hospital stay (LHS) or mean time on mechanical ventilation (MTMV) in patients treated with amantadine or placebo. In this sense, our analysis will add value by the precision of the scales used in the analysis and by being the first study to evaluate hospitalization measures.

We aim to perform a meta-analysis of randomized controlled trials (RCTs) comparing amantadine and placebo on functional improvement in patients with TBI.

## 2 Materials and methods

### 2.1 Eligibility criteria

The inclusion criteria of this meta-analysis was restricted to studies that met all the following eligibility criteria: (1) enrolling patients with traumatic brain injury; (2) comparing amantadine versus placebo; and (3) reporting of at least one outcome of interest. We excluded studies with (1) no control group; (2) no outcomes of interest; (3) non-randomized; and (4) patients without TBI.

### 2.2 Search strategy and data extraction

This systematic review and meta-analysis adhered to the recommendations of the Cochrane Collaboration and the guidelines of the Preferred Reporting Items for Systematic Reviews and Meta-Analyses (PRISMA) statement (15). The protocol was registered on the PROSPERO registry (CRD42024538110). The Cochrane Library, Embase, and PubMed websites were searched. The search included studies published through March 24, 2024, written in English, using the following search terms: “Brain injury,” “Brain trauma,” “TBI,” “Neurostimulant,” “1-aminoadamantane,” “AmantaHCIAZU,” and “PMSAmantadine.” In addition, the references of included studies and systematic reviews were screened for additional relevant studies. The complete search strategy is available in the Supplementary material. Two authors independently extracted the data following predefined search criteria and extracted data to a standardized spreadsheet. Disagreements between these authors were resolved through consensus.

### 2.3 Endpoints and subanalyses

The primary outcomes assessed were Glasgow Coma Scale (GCS), Mini Mental State Examination (MMSE), and DRS, while secondary outcomes included Glasgow Outcome Scale (GOS), mean time on mechanical ventilation (MTMV), and length of hospital stay (LHS). All six outcomes were compared using mean and standard deviation (SD). For DRS, subgroup analyses were performed according to duration of treatment.

### 2.4 Quality assessment

Quality assessment of RCTs was performed using the Cochrane Collaboration’s tool for assessing risk of bias in randomized trials, in which studies are scored as high, low, or unclear risk of bias in 5 domains: selection, performance, detection, attrition, and reporting biases. We planned to investigate publication bias with funnel plot analysis of the primary outcomes.

### 2.5 Statistical analysis

This systematic review and meta-analysis was performed in accordance with the Cochrane Collaboration and the Preferred Reporting Items for Systematic Reviews and Meta-Analysis (PRISMA) statement guidelines (15). Mean differences with a 95% confidence interval was used for continuous comparisons. Heterogeneity was assessed using the Cochrane *Q* test and the Tau statistic and *I*^2^ > 25% considered significant. The leave-one-out test was used to assess potential individual study bias. Given the heterogeneity of countries of origin and differences in protocols, the random effects model was used for all studies. The statistical analysis was performed using the R 4.3.2 software. The effect-size was calculated for DRS and GCS.

## 3 Results

### 3.1 Study selection and characteristics

**Figure 1:**
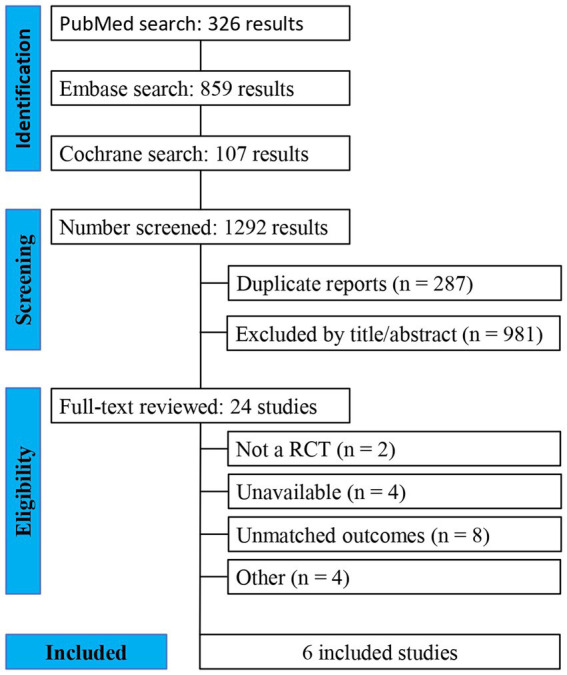
PRISMA flow diagram of study screening and selection.

As detailed in Figure 1, the initial search yielded 1,292 results. After removal of duplicate records and ineligible studies, 24 remained and were fully reviewed based on inclusion criteria. Of these, a total of 6 RCTs were included, comprising 426 patients, 5 of those being parallel RCTs (10, 13, 16–18) and 1 being a randomized cross-over trial (12). A total of 205 (48.12%) patients received amantadine and 221 (51.88%) received placebo. Additional characteristics of the studies are reported in Table 1.

**Table 1:** Baseline characteristics of included studies.

Study	Design	Patients A/P	Age gap	Age^†^, y A/P	Male, % A/P	Treatment protocol	Daily dose^*^	Routes of administration	Measured outcomes
Abbasivash 2019 (17)	RCT	33/33	NA	39.6/35.8	66.7/72.7	Until discharge, death or complication	200 at firsts 3 days / 400 after 3^rd^ day	Oral	GOS LHS MTMV
Ghalaenovi 2018 (10)	RCT	19/21	(16–80)	32.2/41	100/85.7	6 weeks	100 twice daily	NA	DRS GOS MMSE LHS
Giacino 2012 (13)	RCT	87/97	(16–65)	35.5/37.2	74/71	4 weeks	200 at firsts 14 days / 300 until week 3 / 400 until week 4^&^	Oral or nasogastric	DRS CRS-R
Meythaler 2002 (12)	Cross-over RCT	15/20	(16–75)	NA	NA	6 weeks	100 twice daily	NA	DRS GOS MMSE
Shafiee 2022 (18)	RCT	22/22	(15–75)	40.7/47.4	68.2/68.2	8 days	200	Oral	GCS GOS LHS MTMV
Shimia 2023 (16)	RCT	29/28	NA	NA	NA	6 weeks	100 BD for 14 days; 150 BD for 7 days; 200 BD for 21 days.	NA	DRS GOS

^†^mean; ^*^milligrams; ^&^If DRS Score does not fall at least 2 points; A, amantadine; BD, twice a day; NA, not available; P, placebo; RCT, randomized controlled trial; y, years; GCS, Glasgow Coma Scale; LHS, length of hospital stay; MTMV, mean time on mechanical ventilation; DRS, disability rating scale; GOS, Glasgow Outcome Scale; MMSE, Mini-Mental State Examination; CRS-R, Coma Recovery Scale-Revised.

### 3.2 Pooled analysis of all studies

**Figure 2:**
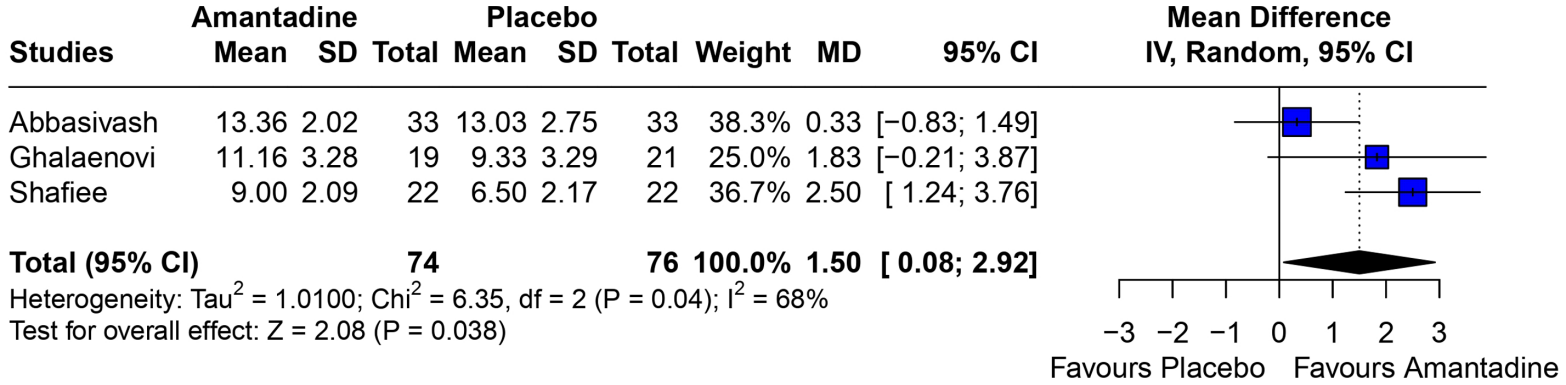
At 7 days, mean GCS was significantly higher in patients receiving amantadine as compared with placebo.

**Figure 3:**
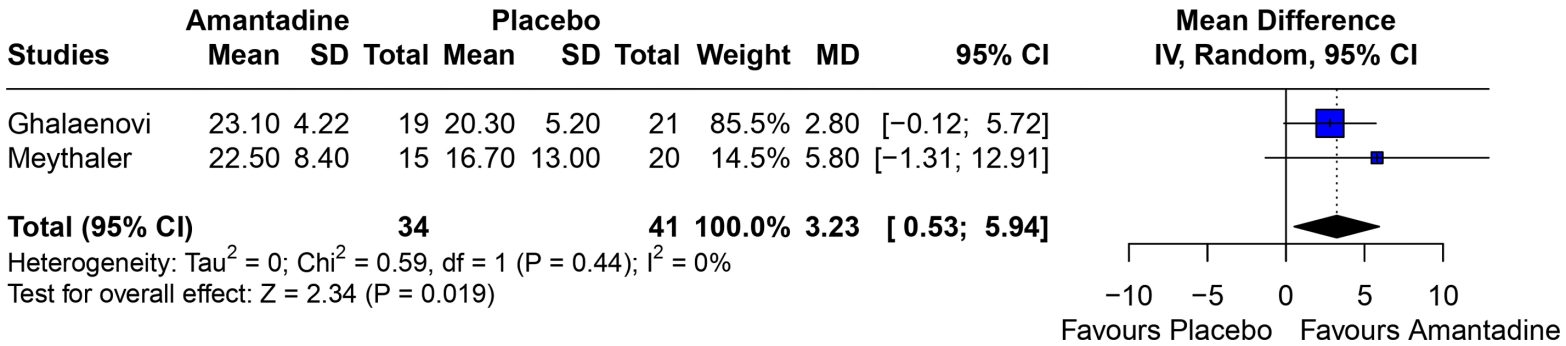
MMSE was significantly higher in patients receiving amantadine as compared with placebo.

**Figure 4:**
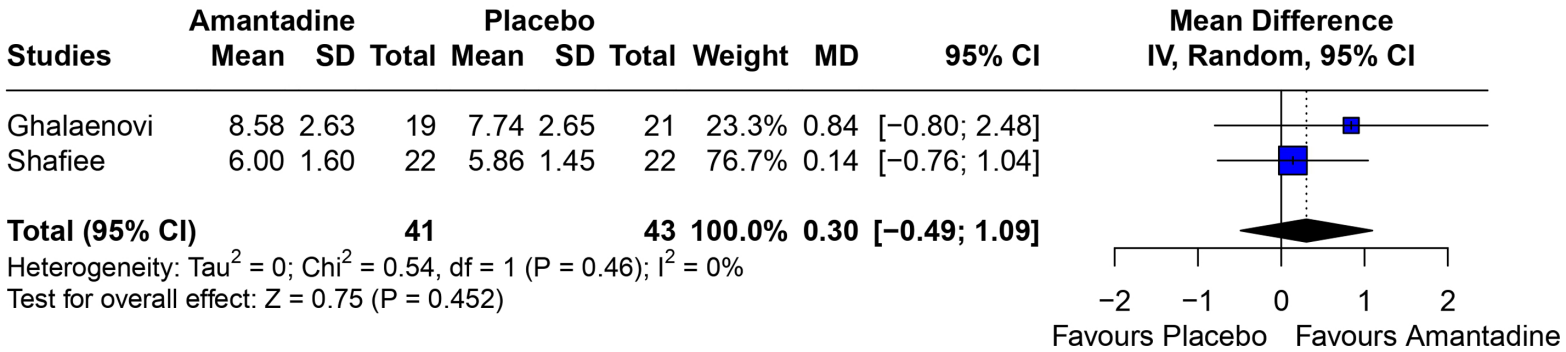
At 3 days, there was no significant difference between groups regarding GCS.

**Figure 5:**
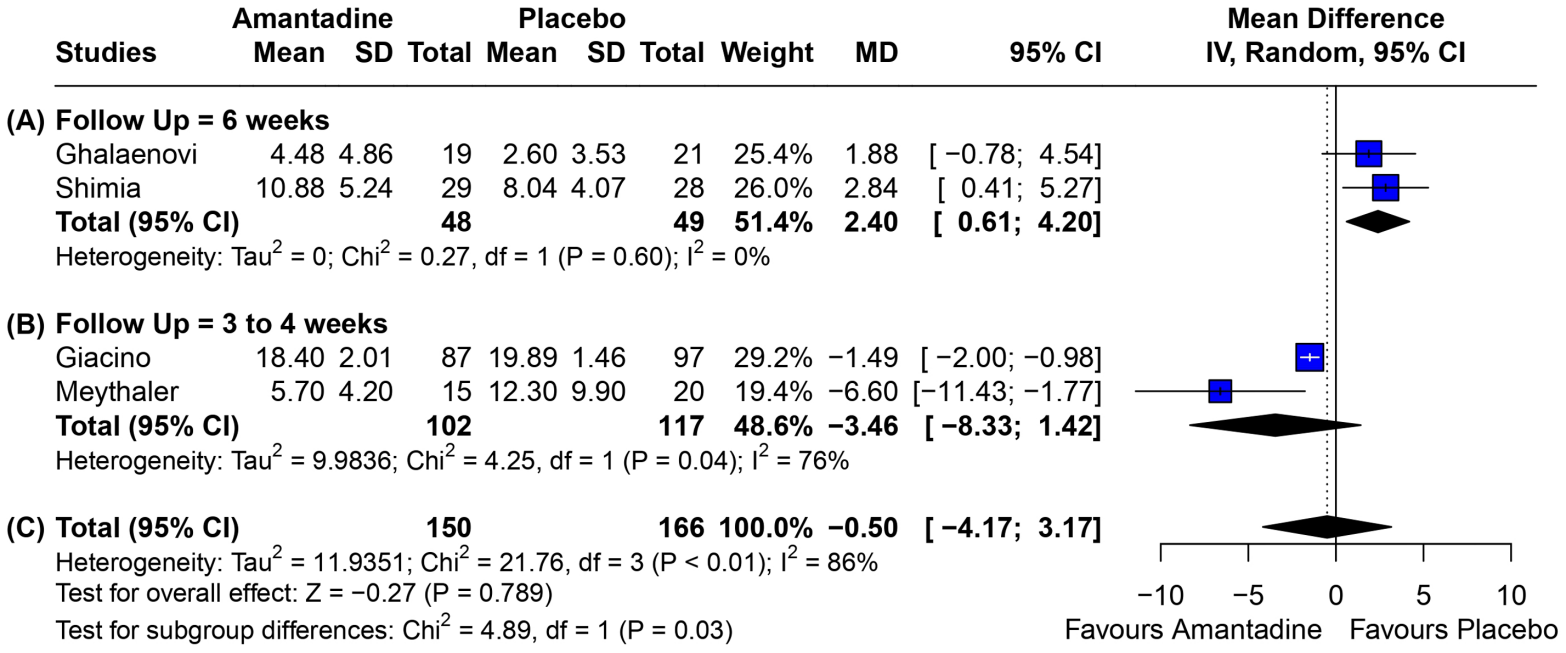
Regarding DRS, **(A)** at 6 weeks of follow-up, there was a significant benefit for patients receiving amantadine; **(B)** at 3–4 weeks of follow-up, there was no difference between groups; and **(C)** on overall analysis regardless of follow-up time, there was no significant difference between patients being treated with amantadine and placebo.

**Figure 6:**
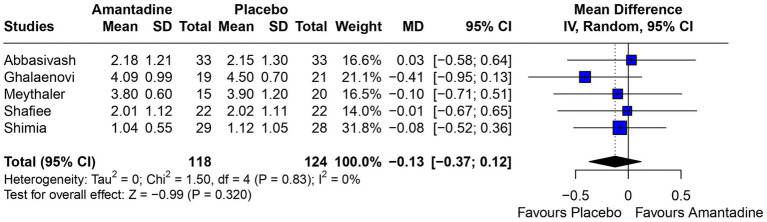
There was no significant difference between groups regarding GOS.

**Figure 7:**
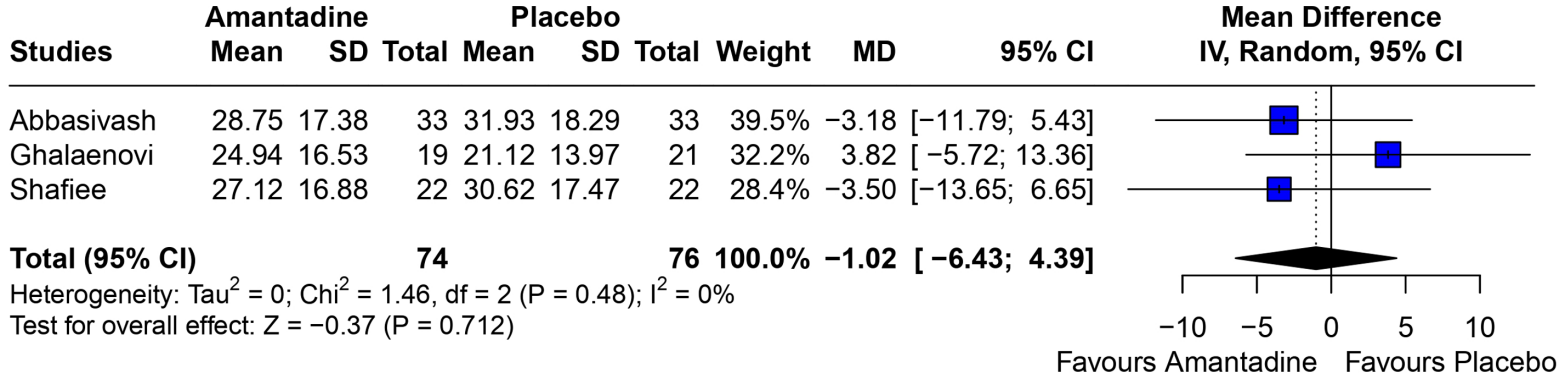
There was no significant difference in the length of hospital stay in patients receiving amantadine versus placebo.

**Figure 8:**
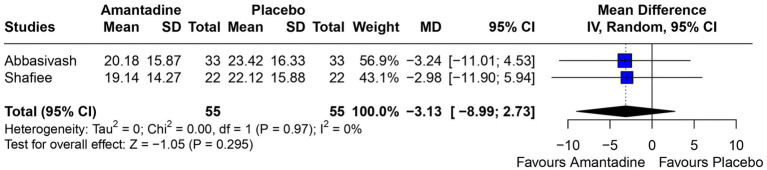
Neither group had a significant advantage in mean time on mechanical ventilation.

Patients receiving amantadine had significantly increased GCS at day 7 (MD 1.50; 95% CI 0.08–2.92; *p* = 0.038; *I*^2^ = 68%; Figure 2) and higher MMSE score (MD 3.23; 95% CI 0.53–5.94; *p* = 0.019; *I*^2^ = 0%; Figure 3). There was no statistically significant difference between groups in terms of GCS at day 3 (MD 0.30; 95% CI -0.49-1.09; *p* = 0.452; *I*^2^ = 0%, Figure 4), DRS (MD −0.50; 95% CI −4.17–3.17; *p* = 0.789; *I*^2^ = 86%, Figure 5) and GOS (MD −0.13; 95% CI −0.37–0.12; *p* = 0.320; *I*^2^ = 0%, Figure 6). The pooled analysis showed no significant difference in hospital length of stay (MD −1.02; 95% CI -6.43-4.39; *p* = 0.712; *I*^2^ = 0%; Figure 7) and mean time on mechanical ventilation (MD −3.13; 95% CI −8.99-2.73; *p* = 0.295; *I*^2^ = 0%; Figure 8) between groups.

### 3.3 Subgroup analysis

A subgroup analysis was performed in the DRS domain in order to analyze the heterogeneity expressed as I^2^ of 86%. For this purpose, the 4 studies used were divided into 2 groups: those whose treatment lasted 3–4 weeks (12, 13) and those whose treatment lasted 6 weeks (10, 16). As result, the DRS analysis between weeks 3 and 4 was not significant and heterogeneity remained high (*I*^2^ = 76%). The analysis at week 6 showed *I*^2^ = 0% and a mean of 2.40 with 95% CI 0.61–4.20. Both results are shown in Figure 5.

### 3.4 Quality assessment

Individual RCT appraisal is reported in Supplementary Figures 1, 2. Two studies were considered as overall “low-risk” of bias (13, 18). Four studies were considered to have “some concerns” for bias in the domain 1 (randomization) and 5 (protocol) (10, 12, 16, 17). The funnel-plot analysis was not performed due to poor sensitivity in the setting of a small number of included studies (<10).

**Figure 9:**
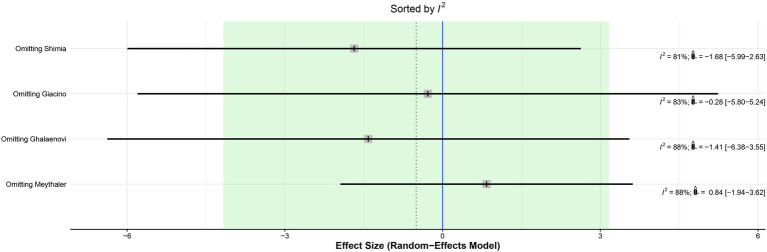
Heterogeneity remained high in all iterations on leave-one-out analysis sorted by *I*^2^ of DRS.

**Figure 10:**
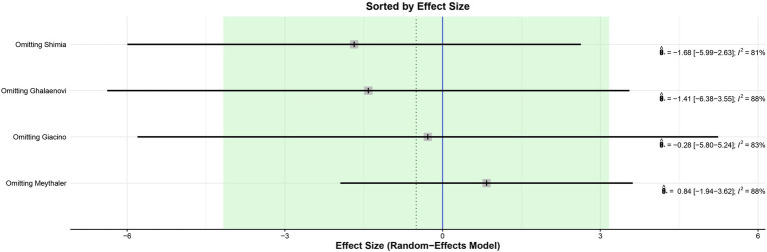
Leave-one-out analysis of DRS sorted by effect size found no significant effect size changes.

**Figure 11:**
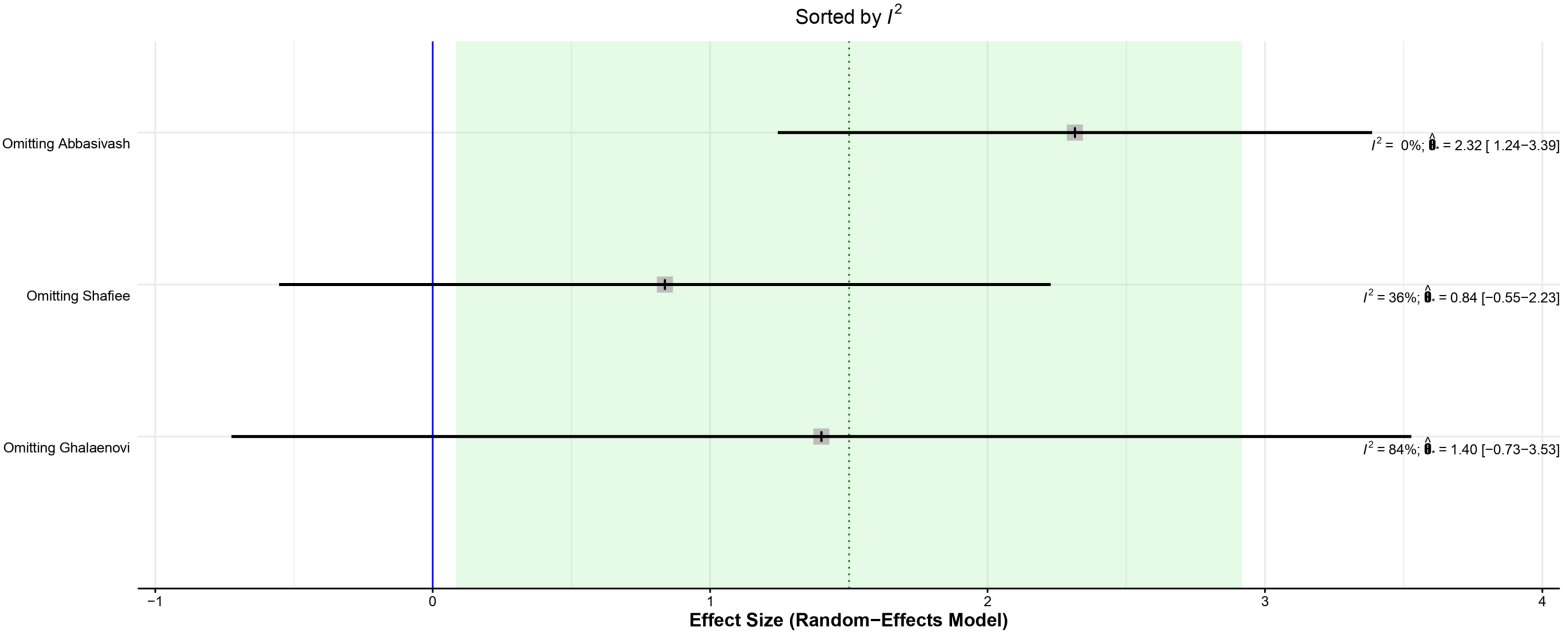
The heterogeneity was reduced to *I*^2^ = 0% when the Abbasivash et al. study was removed.

**Figure 12:**
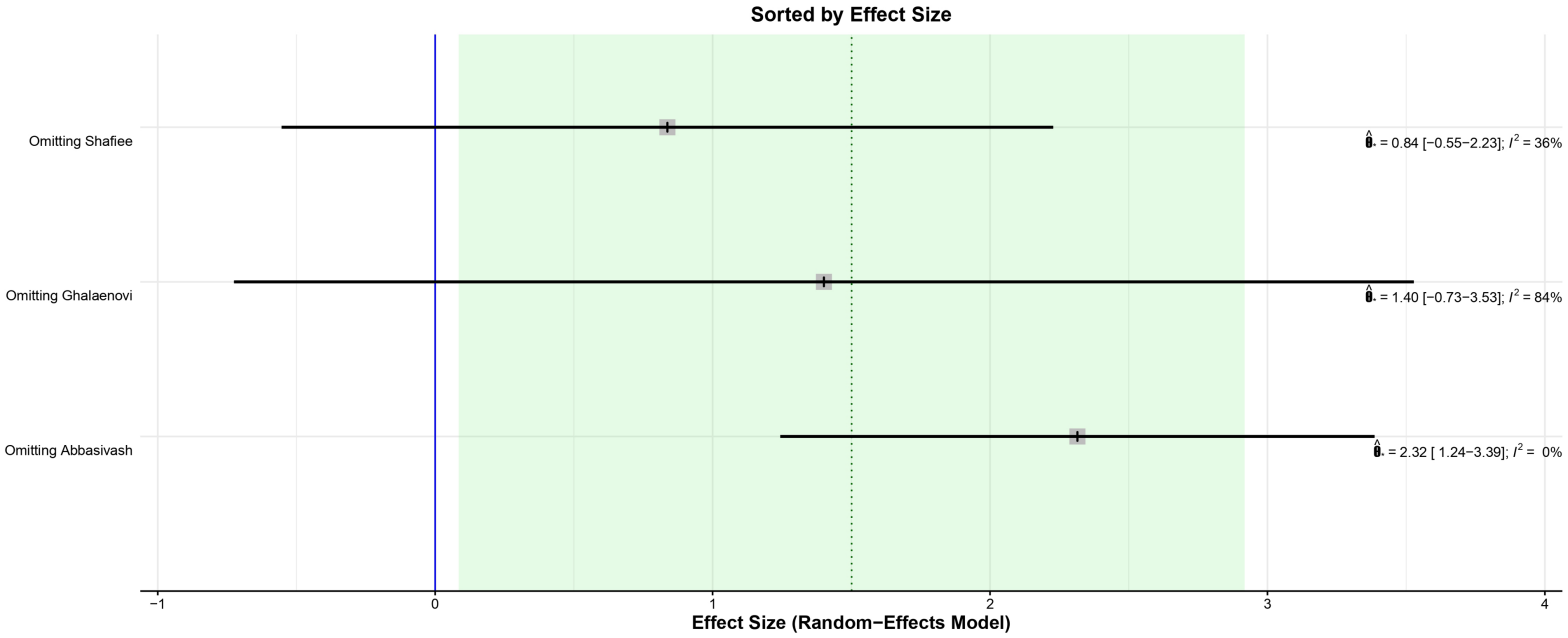
Leave-one-out analysis of GCS at 7 days sorted by effect size showed a statistically significant benefit for patients treated with amantadine when the Abbasivash et al. study was removed.

The leave-one-out analysis of DRS found high heterogeneity within each iteration when sorted by *I*^2^ (Figure 9) and no significant effect size changes were observed (Figure 10). For GCS at 7 days, leave-one-out analysis shown *I*^2^ = 0% (Figure 11) and significantly higher effect size favoring patients being treated with amantadine (Figure 12) when Abbasivash et al. study was removed.

## 4 Discussion

In this systematic review and meta-analysis of 6 studies and 426 patients, we compared amantadine with placebo. Major findings included: (1) significantly increased GCS at 7 days in patients receiving amantadine; (2) higher MMSE score compared to placebo; (3) better DRS between 3rd and 4th week in favor of the placebo group; (4) and no significant differences between groups in terms of GOS, LHS, and MTMV.

A 2022 observational study (19) showed that the use of amantadine significantly improved the GCS of patients with TBI compared to those who did not use it, by more than 3 points at day 5 and at day 10. The GCS is a measure of consciousness used to objectively describe the severity of injury through the patient’s level of consciousness, which is clinically interpreted as the quality of the patient’s motor, eye, and speech responses (20). The scale analyzes aspects of responsiveness to classify the patient from 3 (worst) to 15 (best). In this study, the GCS was analyzed at two time points according to the information available in the primary studies: on day 3 and day 7 after the start of drug therapy. The difference between the results of the amantadine and control groups was not significant on day 3, but was significant on day 7. This result may be explained by the short duration of treatment on day 3, which is probably insufficient for the drug to show significant results, but also by the fact that the analysis was performed using only two studies, in contrast to the GCS on day 7, which was analyzed using three studies and may have been significant due to the longer time elapsed since the start of treatment. This result is probably due to amantadine acting on the areas responsible for regulating wakefulness, activation, and attention. The drug acts by increasing dopamine in the substantia nigra and neurotransmission within the limbic and frontal mesencephalic system of the striatum (13).

Although significant, the GCS on day 7 deserves some consideration. The *I*^2^ of the result was 68%, high for the parameters chosen for this study. A statistical validation was performed through a leave-one-out test using the studies, following the recommendation of Willis et al. (21) by removing one of the studies from the analysis at a time to investigate which study was accountable for the high heterogeneity of the results. When the Abbasivash study was removed, the heterogeneity was reduced to 0%, the test for overall effect yielded a *p*-value <0.0001, and the result remained significant, as shown in Figure 9. To ensure greater robustness to the analysis of GCS on day 7, the data available in the Abbasivash study were included, although they correspond to day 13.73 (mean). This decision may have biased the result due to the time of outcome assessment variability within the primary studies. It must be noted that GCS has been used to assess the level of consciousness, although it does not provide a comprehensive view of overall brain function. In this sense, its use can be influenced not only by the primary clinical condition, but also by associated traumatic injuries, cardiac and/or respiratory complications, and previous conditions. Additionally, the use of sedatives or analgesics during treatment also may alter GCS. These considerations are relevant to all observations based on GCS (22).

The MMSE is used as a diagnostic tool to evaluate cognitive impairment in patients with TBI and studies suggest it might be a good predictor of rehabilitation (23, 24). Retrospective studies on patients with moderate to severe TBI found that MMSE had a correlation of −0.707 with DRS at discharge (24) and a higher Modified Barthel Index after rehabilitation (23). However, contrary to those studies, our analysis have shown a significantly higher MMSE and a higher DRS at 6 weeks follow-up on patients treated with amantadine. Although the treatment duration of the patients included in Meythaler’s study was half that of Ghalaenovi’s study, both analyses tended to favor the amantadine group, and together they form a significant result. In addition, both studies included patients with the same admission GCS (< 10), the sample of both studies was very close, and all other relevant details of the studies were similar, minimizing the bias of this analysis.

An observational study of 124 patients showed that amantadine has the ability to improve DRS (25). In addition, a 2023 study by Tracy et al., with 55 patients, confirmed the benefit of amantadine in DRS (26). Additionally, a previous meta-analyses also found a significant functional improvement in TBI patients treated with amantadine (27). Nonetheless, there was high heterogeneity due to different outcome measures (such as DRS, GCS and GOS) and no subgroup analyses regarding different follow-up time points. The results of our analysis were not consistent with their findings and had high heterogeneity. Although amantadine showed a positive impact on GCS, it seems not to have a significant long-term effect on cognition and functionality. The concept of cognition is broad and involves memory, planning, visual–spatial recognition, and other aspects (28). In our subanalysis, after 6 weeks of follow-up, there was a significant improvement in functionality (as measured by DRS) for patients in the placebo group, indicating that amantadine may be related to worse long-term outcomes regarding cognition and functionality. We hypothesize that although amantadine may accelerate the awakening of patients through the enhancement of dopamine in the substantia nigra and in neurotransmission within the mesencephalic limbic and frontal striatum loop system, this earlier recovery might be associated with a worse cognitive and functional outcome. One of the pathophysiological mechanisms that may be related to this hypothesis is that the blockade of synaptic transmission promoted by NMDA antagonists might hinder neuronal survival at longer follow-up time points, partly due to off-target neurotoxicity and the effects of inhibiting normal neuroplasticity and synaptic function (29–31). Perhaps, this discrepancy can be also explained by the methodological difference between our analysis and the previous studies. In our study, only RCTs were used for the analysis, which have a significantly lower possibility of bias than observational studies. In addition, 4 studies were used for the analysis, which increases the statistical power due to the larger number of patients compared to single observational studies. Moreover, we attempted to explore heterogeneity with a subgroup analysis by time of follow-up and using leave-one-out test. As a result, the subanalysis of studies that treatment lasted for 6 weeks showed a favorable result for the placebo group. However, the subanalysis at weeks 3 and 4 was not significant and heterogeneity remained high. The heterogeneity remained high even within the leave-one-out test. Whyte et al. theorize that the difference in the final outcome of DRS can be explained by the difference in the time it takes to transfer patients to the hospital. Those who were transferred earlier tended to have a better prognosis and leave the hospital sooner. Therefore, the studies with up to 6 weeks of follow-up may have included patients who were admitted later, had a worse prognosis, and therefore stayed in the hospital longer than in the studies with 3–4 weeks of follow-up. Thus, the advantage of the placebo group may actually be due to a bias in the severity of the patients included.

In a previous 11 year propensity matched retrospective analysis comparing use of amantadine or standard treatment in patients with severe traumatic brain injury, patients receiving amantadine were less likely to have favorable recovery (measured by GCS at discharge) and had a longer LHS (32); however, our findings based on RCTs did not find a significant difference between groups in GOS or length of hospital stay. Additionally, the pooled analysis showed no difference in MTMV.

Our study has some limitations. First, a cross-over trial was pooled with non-crossover RCTs to increase the statistical power of the analysis. To mitigate the potential bias of this pooling, the results of the additional cross-over trial were considered only up to the start of the washout period to avoid carry-over effect, even though this shortens treatment duration. Second, the authors did not obtain the full Shimia article and used the information in the abstract as the basis for this study. To minimize the bias of this choice, they used the information from Shimia’s paper that was available from previous studies that had also used it. There was variability within the studies on the severity of TBI and medication dosage, which might partially explain high heterogeneity found on GCS at 7 days and DRS; leave-one-out analysis and subgroup analysis were performed to address this issue. No significant differences were found on leave-one-out analysis sorted by effect size (Figures 11, 12). In addition, systematic reviews have inherent limitations and are susceptible to publication bias, language bias, bias in primary studies, bias from combining studies with relatively different populations, comparison of outcomes, and definition of outcomes.

## 5 Conclusion

This meta-analysis of 426 TBI patients demonstrated the efficacy of amantadine in improving cognition compared with placebo. The improvement in patients’ level of consciousness as measured by the GCS and the MMSE was significantly greater in those who used amantadine compared with placebo. However, the improvement in the DRS between the 3rd and 4th week of treatment was more favorable for patients in the placebo group. Altogether, these findings suggest a good theoretical basis for selecting amantadine as a good drug for reversing cognitive damage after TBI, with the addendum that the drug performs worse on some scales.

## Data Availability

The original contributions presented in the study are included in the article/supplementary material, further inquiries can be directed to the corresponding author.
